# Substance P Antagonism Prevents Chemotherapy-Induced Cardiotoxicity

**DOI:** 10.3390/cancers13071732

**Published:** 2021-04-06

**Authors:** Ashiq Legi, Emma Rodriguez, Thomas K. Eckols, Cyrus Mistry, Prema Robinson

**Affiliations:** 1 Division of Internal Medicine, Department of Infectious Diseases, Infection Control & Employee Health, The University of Texas MD Anderson Cancer Center, Houston, TX 77030-3772, USA; ashiqlegi@gmail.com (A.L.); EERodriguez1@mdanderson.org (E.R.); TkEckols@mdanderson.org (T.K.E.); 2 University of Texas at Dallas, Richardson, TX 75080, USA; Cym190000@utdallas.edu

**Keywords:** substance P, chemotherapy associated cardiotoxicity

## Abstract

**Simple Summary:**

Anthracyclines are a class of chemotherapeutics that are an essential component of many treatment regimens for solid and blood tumors. Doxorubicin (DOX), an anthracycline is broadly considered the most active single agent available for many cancers. However, effective use of anthracyclines is limited due to the possibility of cardiotoxicity, thus causing restrictions on treatment options for treatable cancers. Our studies indicate the SP/NK1R system as a promising novel target and use of NK1R antagonists as a translational tool for prevention of chemotherapy-associated cardiotoxicity in cancer.

**Abstract:**

*Background*: Doxorubicin (DOX), used in chemotherapeutic regimens in many cancers, has been known to induce, cardiotoxicity and life-threatening heart failure or acute coronary syndromes in some patients. We determined the role of Substance P (SP), a neuropeptide and its high affinity receptor, NK-1R in chemotherapy associated cardiotoxicity in mice. We determined if NK-1R antagonism will prevent DOX-induced cardiotoxicity in vivo. *Methods*: C57BL/6 mice (6- week old male) were injected intraperitoneally with DOX (5 mg per kilogram of body weight once a week for 5 weeks) with or without treatment with aprepitant (a NK-1R antagonist, Emend, Merck & Co., Kenilworth, NJ, USA). Five different dosages of aprepitant were administered in the drinking water five days before the first injection of DOX and then continued until the end of the experiment. Each of these 5 doses are as follows; Dose 1 = 0.9 µg/mL, Dose 2 = 1.8 µg/mL, Dose 3 = 3.6 µg/mL, Dose 4 = 7.2 µg/mL, Dose 5 = 14.4 µg/mL. Controls consisted of mice injected with PBS (instead of DOX) with or without aprepitant treatment. The experiment was terminated 5 weeks post-DOX administration and various cardiac functional parameters were determined. Following euthanization, we measured heart weight to body weight ratios and the following in the hearts, of mice treated with and without DOX and aprepitant; (a) levels of SP and NK1R, (b) cardiomyocyte diameter (to determine evidence of cardiomyocyte hypertrophy), (c) Annexin V levels (to determine evidence of cardiac apoptosis), and (d) ratios of reduced glutathione (GSH) to oxidized glutathione (GSSG) (to determine evidence of oxidative stress). *Results*: We demonstrated that the levels of SP and NK1R were significantly increased respectively by 2.07 fold and 1.86 fold in the hearts of mice treated with versus without DOX. We determined that DOX-induced cardiac dysfunction was significantly attenuated by treatment with aprepitant. Cardiac functional parameters such as fractional shortening (FS), ejection fraction (EF) and stroke volume (SV) were respectively decreased by 27.6%, 21.02% and 21.20% compared to the vehicle treated group (All, *p* < 0.05, ANOVA). Importantly, compared to treatment with DOX alone, treatment with lower doses of aprepitant in DOX treated mice significantly reduced the effects of DOX on FS, EF and SV to values not significantly different from sham (vehicle treated) mice (All, *p* < 0.05, ANOVA). The levels of, apoptosis marker (Annexin V), oxidative stress (ratio of GSH with GSSG) and cardiomyocyte hypertrophy were respectively increased by 47.61%, 91.43% and 47.54% in the hearts of mice treated with versus without DOX. Compared to the DOX alone group, treatment with DOX and Dose 1, 2 and 3 of aprepitant significantly decreased the levels of each of these parameters (All *p* < 0.05, ANOVA). Conclusions: Our studies indicate that the SP/NK1-R system is a key mediator that induces, DOX-induced, cardiac dysfunction, cardiac apoptosis, cardiac oxidative stress and cardiomyocyte hypertrophy. These studies implicate that NK-1R antagonists may serve as a novel therapeutic tool for prevention of chemotherapy induced cardiotoxicity in cancer.

## 1. Introduction

Anthracyclines are a class of chemotherapeutics that are an essential component of many treatment regimens for solid and blood tumors [1,2,3,4]. The anthracycline doxorubicin (DOX) is broadly considered the most active single agent available for many cancers [2,5,6]. DOX treatment has however been known to cause cardiotoxicity and cardiac dysfunction leading to heart failure or acute cardiac manifestations in some patients [7,8,9]. Novel agents that attenuate the cardiotoxicity of DOX, are urgently needed.

Substance P (SP), is a neuropeptide and pain transmitter [10]. Nerves, endothelial cells and cells of the immune system make SP and respond to SP [11,12,13,14,15,16]. Substance P (SP) is known to stimulate production of reactive oxygen species (ROS) [17,18]. Elevated cardiac ROS is linked with heart injury/failure in other cardiac settings [19,20]. Our in vitro studies have previously demonstrated that treatment with aprepitant, an NK-1R antagonist prevented DOX-induced cardiomyocyte death [21]. We showed using H9C2, a cardiomyocyte cell line, that aprepitant pretreatment, decreases the cardiomyocyte killing induced by DOX. Furthermore, we had demonstrated that the levels of, ROS and apoptotic cell death were decreased in the H9C2 cells in response to aprepitant treatment [21].

In the current studies, we have determined the role of Substance P (SP), a neuropeptide in chemotherapy associated cardiotoxicity in vivo. We quantitated the levels of SP and NK1R in the hearts of mice treated with and without DOX. We determined if the levels of, apoptosis marker, Annexin V and oxidative stress (as determined by measuring the ratio of GSH to GSSG) within the hearts and cardiomyocyte hypertrophy will be significantly altered in response to DOX treatment. Furthermore, we determined if aprepitant treatment would reverse these DOX-induced alterations. Most importantly, we determined if DOX-induced cardiac dysfunction as determined by echocardiogram (Echo) readings would be attenuated with aprepitant treatment in DOX treated mice.

## 2. Materials and Methods

### 2.1. Murine Model of Doxorubicin Induced Cardiotoxicity

This study was performed in accordance with the recommendations in the Guide for the Care and Use of Laboratory Animals of the National Institutes of Health. The IACUC Committee of the University of Texas MD Anderson Cancer Center approved the protocol (IACUC Protocol number 00001625-RN00, Title: Role of substance P in chemotherapy Induced Cardiotoxicity). All efforts were taken to ameliorate animal suffering. Mice were housed under BSL2 biohazard facility conditions. The mice were observed twice daily for the duration of the experiments. Mice that became moribund were considered to have reached the end of the experiment and were humanely euthanized.

Six week old, male C57BL/6 mice were administered doxorubicin (5 mg per kilogram of body weight, intraperitoneally (i.p).) once a week for 5 weeks. Five days before the first intraperitoneal injection of doxorubicin, mice were administered 5 different doses of aprepitant in drinking water and continued until end of experiment (Dose 1 = 0.9 µg/mL; Dose 2 = 1.8 µg/mL; Dose 3 = 3.6 µg/mL; Dose 4 = 7.2 µg/mL; Dose 5 = 14.4 µg/mL). We then studied heart functions by echocardiographic studies in the different groups on mice. There were a total of 12 mice groups, doxorubicin was dissolved in 1X phosphate buffered saline (PBS) and aprepitant was dissolved in 5% Dimethyl sulfoxide (DMSO) in 1X PBS. The experimental groups were injected with doxorubicin, without or with aprepitant treatment. The control groups were injected with vehicle alone (1X PBS) instead of doxorubicin without or with aprepitant treatment. Echocardiography was performed to determine left ventricular function to determine evidence of cardiac dysfunction as outlined below, following which mice were humanely sacrificed and hearts harvested. Following euthanization, the different parameters measured were; heart-to-body weight ratio, cardiomyocyte diameter, cardiac levels of SP, NK1R, Annexin V and oxidative stress.

### 2.2. Echo Procedure

The small animal imaging facility, a MD Anderson research core was used to perform the Echo procedure. Using a Vevo 2100 ultrasound machine equipped with a 30 MHz transducer (Visualsonics, Toronto, ON, Canada), in vivo cardiac function and morphology were assessed. Mice were anesthetized in an induction chamber using 2.5% isoflurane and then transferred to a heated ECG platform for heart rate monitoring during the imaging procedure. Standard B-mode (2D) and M-mode images were taken in the short axis position at the level of the papillary muscles for each animal. Data analysis for M-mode images were analyzed using the cardiac analysis package and B-mode images were analyzed using Visualsonics VevoStrain software. Results are expressed as the mean values of each Echo readout ± Standard Error of Mean (SEM) for each group.

### 2.3. Measurement of Heart-to-Body Weight Ratio

Briefly, mice were weighed and anesthetized followed by sacrificing by cervical dislocation. The heart was harvested and weighed. The heart weight relative to body weight was calculated. Results are expressed as the mean Hw/Bw ratio ± SEM for each group.

### 2.4. Measurement of Cardiomyocyte Hypertrophy

Evidence of cardiomyocyte hypertrophy was determined by measuring diameter of cardiomyocytes following histopathological processing of the harvested hearts. Briefly, hearts were fixed in 4% paraformaldehyde, embedded in paraffin, sectioned, stained with hematoxylin and eosin, and examined microscopically at 200× magnification. Cardiac hypertrophy was determined by measuring mean cardiomyocyte diameter as determined by measuring the diameter of 50 myocytes using NIH IMAGE v.1.62 software (National Institute of Health, Bethesda, MD, USA). Results are expressed as the mean diameter ± SEM for each group

### 2.5. Measurement of Substance P and NK1R Levels within the Heart

We determined whether DOX treatment increased cardiac SP and NK1R levels. Quantitation of SP protein was performed as described previously [21]. Briefly, hearts derived from the different groups were washed once with cold 1x PBS containing protease inhibitor cocktail (04 693 132 001, Roche, Indianapolis, IN, USA). The hearts were then homogenized in lysis buffer (43-040, Cell Signaling, Danvers, MA, USA) containing protease inhibitor cocktail (04 693 132 001, Roche) and centrifuged at 17,000× *g* for 15 min at 2–8 °C. The SP and NK1R in the supernatant was then respectively quantitated using SP ELISA kit (cat no. ADI-900-018, Enzo Lifesciences, Farmingdale, NY, USA) and the NK1R ELISA kit (cat no. MBS262767, My BioSource, San Diego, CA, USA). Total protein was quantitated using the Bradford method (cat no. 500-0006, Bio-Rad, Hercules, CA, USA). Results are expressed as picograms of SP or NK1R per milligram of total protein ± SEM for each group.

### 2.6. Measurement of Annexin V Levels, An Indicator of Apoptosis within the Heart

Cardiac Annexin V levels were determined in heart tissue lysates using an Annexin V ELISA kit (cat no. BMS252TEN, Invitrogen, Carlsbad, CA, USA) according to the manufacturer’s instructions. Briefly, Annexin V present in the sample or standard bound to antibodies adsorbed to the microwells. A biotin-conjugated anti Annexin V antibody was then added which bound to human Annexin V captured by the first antibody. Following incubation unbound biotin-conjugated anti-Annexin V antibody was removed during a wash step. Streptavidin-HRP was added which bound to the biotin-conjugated anti-Annexin V antibody. Following incubation unbound streptavidin-HRP was removed during a wash step, and substrate solution reactive with HRP was added to the wells. The plates were then read at 450 nm using a spectrophotometer. Annexin V levels were normalized to protein concentrations (as determined by the Bradford method (cat no. 500-0006, Bio-Rad). Annexin V levels are expressed as the mean ± SEM (pg/mg total protein) for each group.

### 2.7. Measurement of GSH to GSSG Ratio, an Indicator of Oxidative Stress within the Heart

Cardiac glutathione (GSH) levels were assayed from heart tissue lysates using a GSH/glutathione disulfide (GSSG) Ratio Detection Kit II (Fluorometric-Green, Abcam 205811; Cambridge, UK) according to the manufacturer’s instructions, and the wavelength was determined (Ex/Em = 490/520 nm). Briefly, the hearts were homogenized in lysis buffer (43-040, Cell Signaling) containing protease inhibitor cocktail (04 693 132 001, Roche) and centrifuged at 17,000× *g* for 15 min at 2–8 °C. The supernatant containing peptides and proteins was recovered. Since fluorometric measurement of GSH requires previous deproteinization, the supernatant was deproteinized using the deproteinizing preparation kit (ab 204708, Abcam). Protein content of all samples was measured prior to deproteinization by the Bradford method (cat no. 500-0006, Bio-Rad) and GSH levels were normalized to protein concentrations. The GSH/Total GSH ratio is expressed as the mean ± SEM for each group.

### 2.8. Statistics

Statistical differences between groups were determined using one-way ANOVA (followed by a pairwise comparison using Tukey’s or Dunn’s test). Significance was set at *p* < 0.05. Statistical analyses were performed with GraphPad Prism 7.03 (San Diego, CA, USA).

## 3. Results

### 3.1. Effect of Doxorubicin on Heart SP and NK1R Protein Levels

**Figure 1:**
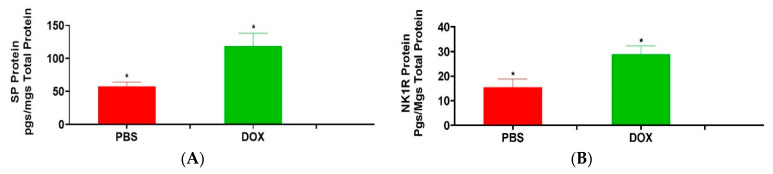
Doxorubicin treatment induces significant increases in the cardiac levels of, substance P and its high affinity receptor, NK1R. Effect of doxorubicin treatment on (**A**) SP levels and (**B**) NK1R levels in the heart. Both SP and NK1R levels were significantly increased in the hearts of DOX treated mice compared to that of the vehicle treated group (*, both *p* < 0.05, Student’s *t*-test).

We determined the contribution of SP and NK1R in induction of DOX-induced cardiotoxicity, we measured SP and NK1R protein levels within the hearts of DOX treated group and compared it to that of the vehicle treated group. We determined that both SP and NK1R were significantly increased respectively by 2.07-fold and 1.86-fold in the hearts of mice treated with versus without DOX. Compared to the vehicle treated group, SP protein levels was significantly increased in the DOX treated group, (Figure 1A; DOX; 119 ± 19.3 pg/mg, *n* = 3 vs. vehicle; 57.4 ± 6.7, *n* = 3: *p* = 0.047, Student’s *t*-test). Similarly, the NK1R protein levels were also significantly increased in the DOX treated group compared to the vehicle treated group, (Figure 1B; DOX; 28.79 ± 3.4 pg/mg, *n* = 3 vs. vehicle; 15.47 ± 3.3, *n* = 3: *p* = 0.0252, Student’s *t*-test).

### 3.2. Effect of Aprepitant on Doxorubicin Induced Changes in Heart Weight to Body Weight Ratios

**Figure 2:**
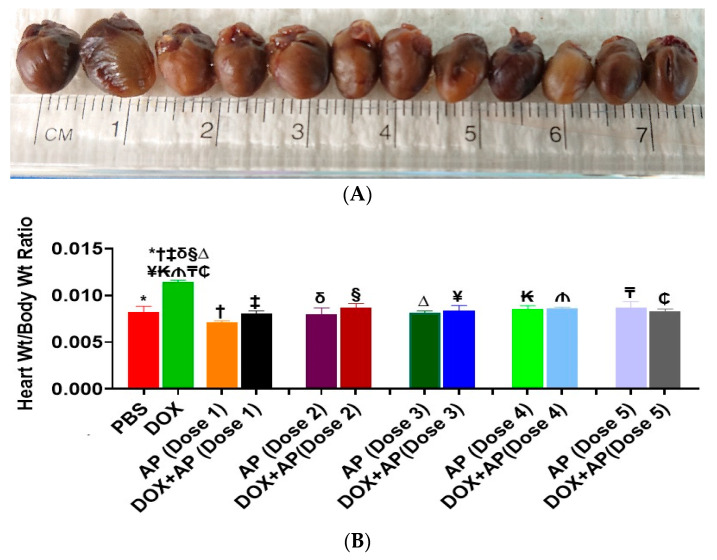
Substance P receptor antagonism significantly attenuates doxorubicin-induced increases in heart size and heart-to-body weight ratios. (**A**) Effect of SP receptor antagonism on doxorubicin induced increases in heart size. Gross appearance of a representative heart derived from mice treated with the following, order from left to right; Sham, DOX alone, Dose1 of aprepitant (AP) without DOX, DOX+Dose 1 (AP), Dose 2 (AP) without DOX, DOX+Dose 2 (AP) Dose 3 (AP) without DOX, DOX+Dose 3 (AP), Dose4 (AP) without DOX, DOX+Dose 4 (AP), Dose 5 (AP) without DOX, or DOX+Dose 5 (AP).(**B**) Effect of SP receptor antagonism on DOX induced increases in Heart Weight to body weight ratios (Hw/Bw). Heart weight to body weight ratio was significantly increased in the DOX treated group compared to the vehicle treated group (*, *p* < 0.05, ANOVA, Tukey’s test). Importantly, compared to treatment with DOX alone, treatment with all 5 doses of aprepitant (a SP receptor antagonist, Merck & Co.) resulted in significant reduction of the Hw/Bw ratios (*, PBS vs. DOX; **†**, AP (Dose1) vs. DOX; **‡**, DOX+ AP (Dose 1) vs. DOX; **δ**, AP (Dose 2) vs. DOX; **§**, DOX+ AP (Dose 2) vs. DOX; **∆**, AP (Dose 3) vs. DOX; **¥**, DOX+ AP (Dose 3) vs. DOX; **₭**, AP (Dose 4) vs. DOX; **₼**, DOX+ AP (Dose 4) vs. DOX; **₸**, AP (Dose 5) vs. DOX; **₵**, DOX + AP (Dose 5) vs. DOX). ALL *p* < 0.05, ANOVA, Tukey’s test.

There were no significant differences in the body weight of animals between the different groups (results not shown). The heart size (Figure 2A) and the mean heart-to-body weight ratio (Hw/Bw; expressed in terms of mg/g) was significantly increased in the DOX treated group compared to the vehicle treated group, (Figure 2B; DOX; 0.01142 ± 0.0002, *n* = 5 vs. vehicle; 0.0082 ± 0.0006, *n* = 4: *p* = 0.001, ANOVA with Tukey’s post hoc test. Importantly, treatment with all 5 doses of aprepitant by themselves or in conjunction with DOX treated mice significantly reduced the effects of DOX on Hw/Bw ratios to values not significantly different from vehicle treated control mice. Results of only Dose 2 and Dose 3 detailed as follows; DOX+AP (Dose 2); 0.0086 ± 0.0004 mg/kg, *n* = 5 and DOX+AP (Dose 3); 0.0084 ± 0.0005 mg/kg, *n* = 5 vs. DOX; 0.01142 ± 0.0002 mg/kg, *n* = 5: *p* < 0.05 both, ANOVA and Tukey’s test (Figure 2B).

### 3.3. Effect of Aprepitant on Doxorubicin Induced Cardiomyocyte Hypertrophy

**Figure 3:**
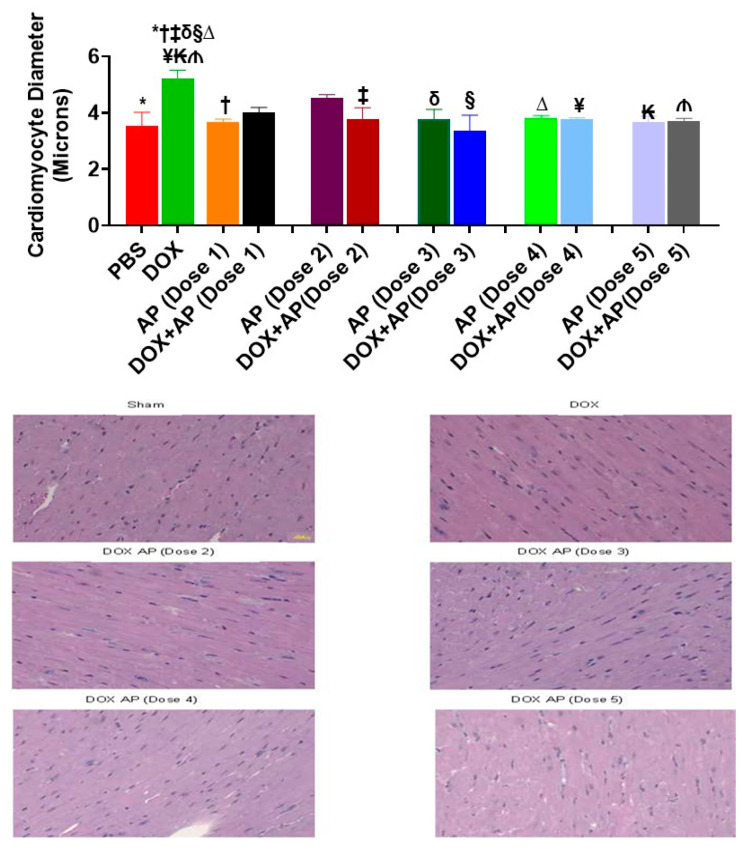
Substance P receptor antagonism significantly attenuates doxorubicin induced cardiomyocyte hypertrophy. Effect of SP receptor antagonism on doxorubicin induced increases in cardiomyocyte hypertrophy. Compared to the vehicle treated group, the cardiomyocyte diameter was significantly increased in response to DOX (*, *p* < 0.05, ANOVA). Importantly, treatment with one or more Doses of aprepitant attenuated the DOX-induced increases in cardiomyocyte hypertrophy. (*, PBS vs. DOX; **†**, AP (Dose1) vs. DOX; **‡,** DOX+ AP (Dose 2) vs. DOX; **δ**, AP (Dose 3) vs. DOX; **§**, DOX+ AP (Dose 3) vs. DOX; **∆**, AP (Dose 4) vs. DOX; **¥**, DOX+ AP (Dose 4) vs. DOX; **₭**, AP (Dose 5) vs. DOX; **₼**, DOX+ AP (Dose 5) vs. DOX; ALL *p* < 0.05, ANOVA Tukey’s test). Bottom Panel: Representative photomicrographs from each of the following groups, Sham, DOX alone (depicting cardiomyocyte hypertrophy), DOX+AP (Dose 2–5, depicting cardiomyocyte sizes to values not significantly different from sham (Magnification 200×)

Compared to the sham control (vehicle treated group), the mean cardiomyocyte diameter (expressed in terms of microns) was significantly increased in response to DOX (Figure 3; DOX; 5.22 ± 0.28 µm, *n* = 4 vs. vehicle; 3.54 ± 0.47 µm, *n* = 2: *p* = 0.03, ANOVA with Tukey’s post hoc test). Importantly, all 5 Dose of aprepitant attenuated the DOX-induced increases of cardiomyocyte diameter as follows; DOX+AP (Dose 1); 4.015 ± 0.17 µm, *n* = 3, DOX+AP (Dose 2); 3.75 ± 0.42 µm, *n* = 3, DOX+AP (Dose 3); 3.35 ± 0.56 µm, *n* = 3, DOX+AP (Dose 4); 3.75 ± 0.07 µm, *n* = 3, DOX+AP (Dose 5); 3.70 ± 0.097 µm, *n* = 3, vs. DOX; 5.22 ± 0.28 µm, *n* = 4: *p* < 0.05 all, ANOVA and Tukey’s test (Figure 3).

### 3.4. Effect of Aprepitant on Doxorubicin Induced Changes in Heart Functions

Echo cardiographic readings was used to determine various cardiac functional parameters such as fractional shortening, ejection fraction, stroke volume, systolic and diastolic diameter and volume. We did not determine any significant differences in the systolic and diastolic, diameter and volume between the groups. However, we demonstrated the ability of aprepitant to reverse many of the heart function parameters that were derogatorily affected by doxorubicin as follows:

#### 3.4.1. Fractional Shortening (FS)

**Figure 4:**
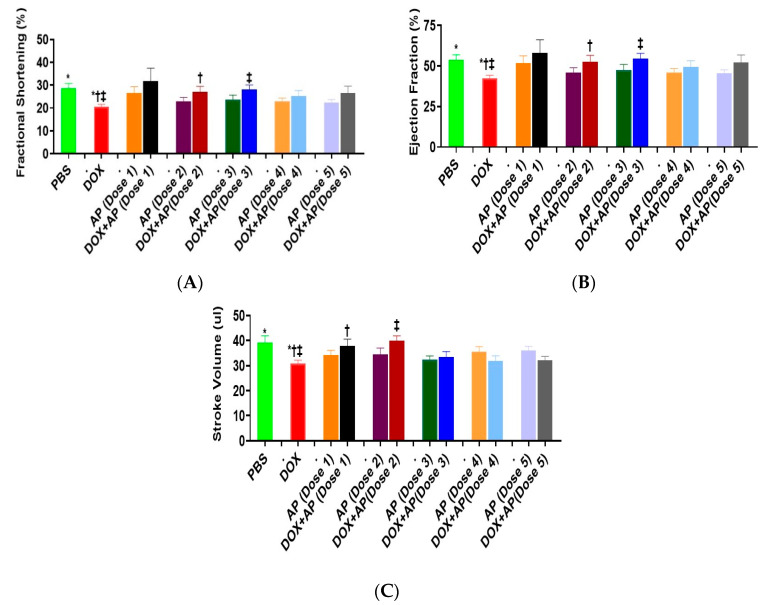
Substance P receptor antagonism significantly attenuates doxorubicin induced cardiac dysfunction; including effects on (**A**) Fractional Shortening (**B**) Ejection Fraction and (**C**) Stroke volume; (A and B; *, PBS vs. DOX; **†**, DOX+ AP (Dose 2) vs. DOX **‡**, DOX + AP (Dose 3) vs. DOX) and (C; *, PBS vs. DOX; **†**, DOX + AP (Dose1) vs. DOX; **‡**, DOX + AP (Dose 2) vs. DOX) ALL *p* < 0.05, ANOVA Tukey’s test

Compared to the vehicle treated group, the mean FS was significantly decreased in response to DOX (Figure 4A; DOX; 20.66 ± 0.9913%, *n* = 9 vs. vehicle; 28.55 ± 2.174%, *n* = 9: *p* = 0.02, ANOVA with Tukey’s post hoc test). Importantly, treatment with Dose 2 and Dose 3 of aprepitant attenuated the effects of DOX on FS; DOX + AP (Dose 2); 27.03 ± 2.443%, *n* = 5 and DOX + AP (Dose 3); 28.10 ± 1.970%, *n* = 5 vs. DOX; 20.66 ± 0.9913%, *n* = 9: *p* < 0.05 both, ANOVA and Tukey’s test (Figure 4A).

#### 3.4.2. Ejection Fraction (EF)

Compared to the vehicle treated group, the mean EF was significantly decreased in response to DOX (Figure 4B; DOX; 42.48 ± 1.788%, *n* = 9 vs. vehicle; 53.79 ± 3.167%, *n* = 9: *p* = 0.03, ANOVA with Tukey’s post hoc test). Importantly, treatment with Dose 2 and Dose 3 of aprepitant attenuated the effects of DOX on EF; DOX + AP (Dose 2); 52.74 ± 3.795%, *n* = 5 and DOX+AP (Dose 3); 54.61 ± 3.148%, *n* = 5 vs. DOX; 42.48 ± 1.788%, *n* = 9: *p* < 0.05 both, ANOVA and Tukey’s test (Figure 4B).

#### 3.4.3. Stroke Volume

Compared to the vehicle treated group, the mean stroke volume was significantly decreased in response to DOX (Figure 4C; DOX; 30.92 ± 1.248 μL *n* = 9 vs. vehicle; 39.24 ± 2.746 μL, *n* = 9: *p* = 0.02, ANOVA with Tukey’s post hoc test). Importantly, treatment with Dose 1 and Dose 2 of aprepitant attenuated the effects of DOX on stroke volume; DOX + AP (Dose 1); 37.95 ± 2.637, *n* = 6 and DOX+AP (Dose 2); 39.84 ± 2.063 μL, *n* = 5 vs. DOX; 30.92 ± 1.248 μL, *n* = 9: *p* < 0.05 both, ANOVA and Tukey’s test (Figure 4C)

### 3.5. Effect of Aprepitant on Doxorubicin Induced Cardiomyocyte Apoptosis

**Figure 5:**
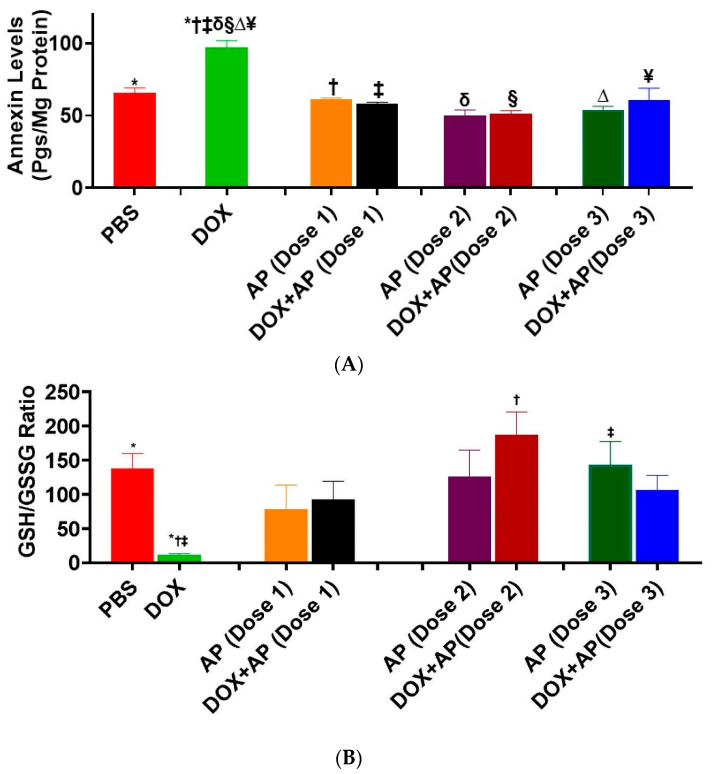
SP receptor antagonism significantly attenuates doxorubicin-induced alterations in (**A**) annexin V levels (apoptosis marker) (*, PBS vs. DOX; **†**, AP (Dose1) vs. DOX; **‡**, DOX + AP (Dose 1) vs. DOX; **δ**, AP (Dose 2) vs. DOX; **§**, DOX + AP (Dose 2) vs. DOX; **∆**, AP (Dose 3) vs. DOX; **¥**, DOX + AP (Dose 3) vs. DOX; **₭**, AP (Dose 4) vs. DOX; **₼**, DOX + AP (Dose 4) vs. DOX; **₸**, AP (Dose 5) vs. DOX; **₵**, DOX + AP (Dose 5) vs. DOX). ALL *p* < 0.05, ANOVA Tukey’s test. SP receptor antagonism significantly attenuates doxorubicin-induced alterations in (**B**) GSH/GSSG ratios (Lower levels of GSS/GSSG ratios indicate oxidative stress) (*, PBS vs. DOX; **†**, DOX + AP (Dose2) vs. DOX; **‡**, AP (Dose 3) vs. DOX). ALL *p* < 0.05, ANOVA Tukey’s test.

The levels of, apoptosis marker (Annexin V) was significantly increased in response to DOX treatment (Figure 5; DOX; 97.28 ± 4.36 pgs/mg, *n* = 2 vs. vehicle; 65.9 ± 3.1 pgs/mg, *n* = 3: *p* = 0.005, ANOVA with Tukey’s post hoc test. Importantly, treatment with Dose 1, Dose 2 and Dose 3 of aprepitant in DOX treated mice significantly reduced the effects of DOX on annexin levels to values not significantly different from vehicle treated control mice; DOX + AP (Dose 1); 58.24 ± 0.98 pgs/mg, *n* = 3, DOX + AP (Dose 2); 51.04 ± 2.44 pgs/mg, *n* = 3 and DOX+AP (Dose 3); 54.08 ± 2.35 pgs/mg *n* = 3 vs. DOX; 97.28 ± 4.36 pgs/mg, *n* = 2: *p* < 0.05 all, ANOVA and Tukey’s test (Figure 5A).

### 3.6. Effect of Aprepitant on Doxorubicin Induced Oxidative Stress

The levels of, GSH to GSSG ratio (with low levels depicting oxidative stress) was significantly decreased in response to DOX treatment (Figure 5B; DOX; 11.36 ± 1.59, *n* = 3 vs. vehicle; 137.7 ± 22.23, *n* = 3: *p* = 0.03, ANOVA with Tukey’s post hoc test. Importantly, treatment with Dose 2 of aprepitant in DOX treated mice significantly reduced the effects of DOX on GSH to GSSG ratios to levels not significantly different from sham (vehicle treated) mice; DOX + AP (Dose 2); 187 ± 33.8, *n* = 3 vs. DOX; 11.36 ± 1.59, *n* = 3: *p* < 0.003, ANOVA and Tukey’s test (Figure 5B).

## 4. Discussion

We determined the role of SP in the pathogenesis of anthracycline-induced cardiotoxicity. We demonstrated that both SP and its high affinity receptor, NK1R were increased in hearts of DOX treated mice. We determined that DOX-induced increases in SP and NK1R levels were accompanied by several manifestations such as increased, Hw/Bw ratios, cardiomyocyte diameter and compensatory hypertrophy. Importantly, these DOX-induced cardiac manifestations were significantly attenuated in response to aprepitant treatment indicating that SP/NK1R pathway is one of the key mediators responsible for induction of DOX induced cardiotoxicity.

We speculate that the heart enlargement in the DOX treated mice may be due to compensatory cardiomyocyte hypertrophy induced because of DOX-induced apoptosis. We did not see any evidence of inflammatory influx in the heart in response to DOX; hence do not attribute inflammation to play a part in DOX-induced heart enlargement.

The cardiomyocyte apoptosis may be probably induced by oxidative stress. We could not determine the levels of reactive oxygen species (ROS) in fresh hearts due to practical considerations. ROS determinations need to be done immediately upon collection of cardiac tissues from the animal, and due to the need for collection of tissues for several other assays from a large number of animals quickly at termination point, it is impractical to perform ROS determinations simultaneously from each heart upon collection. However, determinations of oxidative stress can be performed on frozen tissues; therefore, we elected to perform this assay.

Most importantly, we determined that aprepitant treatment significantly attenuated DOX-induced effects on cardiac functional parameters such as FS, EF and SV.

We and others have determined that SP is detrimental in several heart related pathophysiological conditions. Our studies have previously determined that infection with encephalomyocarditis virus, a causative agent of viral myocarditis, resulted in significantly increased levels of SP [22] We had also demonstrated that mice deficient in either the SP/NK1R mice had significantly decreased EMCV induced manifestations [22,23]. Other groups have shown that SP is involved in inducing chronic volume overload-induced heart failure and deletion of the SP gene protected mice from developing left ventricular hypertrophy [24]. Other studies have demonstrated that magnesium deficiency to result in elevated levels of SP in cardiac lesions [25]. Further studies also demonstrated that NK-1R blockade in the magnesium deficient animals led to attenuation of ROS levels in cardiac cells with resultant improvement in cardiac dysfunction [25,26]. These preceding studies along with or current studies highlight elevated SP levels to be derogatory for the heart and also highlight that inhibition of the SP/NK1R pathway via NK-1R antagonists to be beneficial to treat these pathophysiological cardiac manifestations.

Importantly several studies done by Covenas and Munoz have demonstrated the involvement of the substance P (SP)/neurokinin-1 receptor (NK-1R) system in cancer [27,28,29,30,31,32,33,34]. Aprepitant, a non-peptide NK-1R antagonist, is currently used in clinical practice as antiemetic and also known to show antitumor effects against a broad-spectrum of cancers [27]. Our studies demonstrating aprepitant to protect against chemotherapy associated cardiotoxicity along with other studies that have demonstrated its antitumor effects implicates that aprepitant could be used as a double-edged sword. It could be used as an intelligent bullet against cancer while at the same time protecting against the harmful effects of chemotherapy-associated cardiotoxicity.

DOX, a potent anthracycline has been used for decades for the treatment of many adult and pediatric cancers including breast cancer, leukemia, lymphoma, Hodgkin’s disease, and sarcoma. The continuous use of this anthracycline can lead to life threatening cardiotoxicity, that can manifest in cancer patients years after they have stopped chemotherapy [35]. It has been documented that higher rates of cardiotoxicity are typically observed in elderly patients or persons with pre-existing cardiovascular disease [36]. Effective use of anthracyclines is thus limited due to the possibility of cardiotoxicity, thus causing restrictions on treatment options for treatable cancers. Studies are urgently warranted to determine novel targets for the sole purpose of lifting the restrictions of use of the highly effective anthracycline therapy in cancer.

In the current studies, we have determined that SP/NK1R system is one of a key system responsible for inducing DOX induced cardiotoxicity. We determined that SP receptor antagonism attenuates DOX-induced, cardiac dysfunction, cardiac apoptosis, cardiac oxidative stress and cardiomyocyte hypertrophy. Our studies may indicate the SP/NK1R system as a promising novel target and use of NK1R antagonists as a translational tool for prevention of chemotherapy-associated cardiotoxicity in cancer.

## 5. Conclusions

Our studies indicate that the SP/NK1-R system is a key mediator that induces, DOX-induced, cardiac dysfunction, cardiac apoptosis, cardiac oxidative stress and cardiomyocyte hypertrophy. These studies implicate that NK-1R antagonists may serve as a novel therapeutic tool for prevention of chemotherapy induced cardiotoxicity in cancer.

## References

[1] Lipshultz S.E., Colan S.D., Gelber R.D., Perez-Atayde A.R., Sallan S.E., Sanders S.P. (1991). Late cardiac effects of doxorubicin therapy for acute lymphoblastic leukemia in childhood. N. Engl. J. Med..

[2] Sinha B.K., Mimnaugh E.G., Rajagopalan S., Myers C.E. (1989). Adriamycin activation and oxygen free radical formation in human breast tumor cells: Protective role of glutathione peroxidase in adriamycin resistance. Cancer Res..

[3] Young R.J., Natukunda A., Litiere S., Woll P.J., Wardelmann E., van der Graaf W.T. (2014). First-line anthracycline-based chemotherapy for angiosarcoma and other soft tissue sarcoma subtypes: Pooled analysis of eleven European organisation for research and treatment of cancer soft tissue and bone sarcoma group trials. Eur. J. Cancer.

[4] Novakova Z., Stastna J., Honzikova K., Hrstkova H., Honzikova N., Zavodna E., Fišer B., Honzík P. (2010). Anthracycline therapy and 24-hour blood-pressure profile in long-term survivors of childhood cancer. Physiol. Res..

[5] Tassone P., Tagliaferri P., Perricelli A., Blotta S., Quaresima B., Martelli M.L., Goel A., Barbieri V., Costanzo F., Boland C.R. (2003). BRCA1 expression modulates chemosensitivity of BRCA1-defective HCC1937 human breast cancer cells. Br. J. Cancer.

[6] Curigliano G., Cardinale D., Suter T., Plataniotis G., de Azambuja E., Sandri M.T., Criscitiello C., Goldhirsch A., Cipolla C., Roila F. (2012). Cardiovascular toxicity induced by chemotherapy, targeted agents and radiotherapy: ESMO clinical practice guidelines. Ann. Oncol..

[7] Yeh E.T., Tong A.T., Lenihan D.J., Yusuf S.W., Swafford J., Champion C., Durand J., Gibbs H., Zafarmand A.A., Ewer M.S. (2004). Cardiovascular complications of cancer therapy: Diagnosis, pathogenesis, and management. Circulation.

[8] Smith L.A., Cornelius V.R., Plummer C.J., Levitt G., Verrill M., Canney P., Jones A. (2010). Cardiotoxicity of anthracycline agents for the treatment of cancer: Systematic review and meta-analysis of randomised controlled trials. BMC Cancer.

[9] Von Hoff D.D., Layard M.W., Basa P., Davis H.L., Von Hoff A.L., Rozencweig M., Muggia F.M. (1979). Risk factors for doxorubicin-induced congestive heart failure. Ann. Intern. Med..

[10] Honore P., Rogers S.D., Schwei M.J., Salak-Johnson J.L., Luger N.M., Sabino M.C., Clohisy D., Mantyh P. (2000). Murine models of inflammatory, neuropathic and cancer pain each generates a unique set of neurochemical changes in the spinal cord and sensory neurons. Neuroscience.

[11] Weinstock J.V., Blum A., Walder J., Walder R. (1988). Eosinophils from granulomas in murine schistosomiasis mansoni produce substance P. J. Immunol..

[12] Maggi C.A. (1997). The effects of tachykinins on inflammatory and immune cells. Regul. Pept..

[13] Ho W.Z., Lai J.P., Zhu X.H., Uvaydova M., Douglas S.D. (1997). Human monocytes and macrophages express substance P and neurokinin-1 receptor. J. Immunol..

[14] Church D., Arkinstall S.J., Vallotton M.B., Chollet A., Kawashima E., Lang U. (1996). Stimulation of atrial natriuretic peptide release by neurokinins in neonatal rat ventricular cardiomyocytes. Am. J. Physiol..

[15] Goode T., O'Connell J., Sternini C., Anton P., Wong H., O'Sullivan G.C., Collins J.K., Shanahan F. (1998). Substance P (neurokinin-1) receptor is a marker of human mucosal but not peripheral mononuclear cells: Molecular quantitation and localization. J. Immunol..

[16] Cook G.A., Elliott D., Metwali A., Blum A.M., Sandor M., Lynch R., Weinstock J.V. (1994). Molecular evidence that granuloma T lymphocytes in murine schistosomiasis mansoni express an authentic substance P (NK-1) receptor. J. Immunol..

[17] Tejero-Taldo M.I., Kramer J.H., Mak Iu T., Komarov A.M., Weglicki W.B. (2006). The nerve-heart connection in the pro-oxidant response to Mg-deficiency. Heart Fail Rev..

[18] Sterner-Kock A., Braun R.K., van der Vliet A., Schrenzel M.D., McDonald R.J., Kabbur M.B., Vulliet P.R., Hyde D.M. (1999). Substance P primes the formation of hydrogen peroxide and nitric oxide in human neutrophils. J. Leukoc. Biol..

[19] Chen K., Keaney J.F. (2012). Evolving concepts of oxidative stress and reactive oxygen species in cardiovascular disease. Curr. Atheroscler Rep..

[20] Sugamura K., Keaney J.F. (2011). Reactive oxygen species in cardiovascular disease. Free Radic. Biol. Med..

[21] Robinson P., Kasembeli M., Bharadwaj U., Engineer N., Eckols K.T., Tweardy D.J. (2016). Substance P receptor signaling mediates doxorubicin-induced cardiomyocyte apoptosis and triple-negative breast cancer chemoresistance. Biomed Res. Int..

[22] Robinson P., Garza A., Moore J., Eckols T.K., Parti S., Balaji V., Vallejo J., Tweardy D.J. (2009). Substance P is required for the pathogenesis of EMCV infection in mice. Int. J. Clin. Exp. Med..

[23] Robinson P., Taffet G.E., Engineer N., Khumbatta M., Firozgary B., Reynolds C., Pham T., Bulsara T., Firozgary G. (2015). Substance P receptor antagonism: A potential novel treatment option for viral-myocarditis. Biomed Res. Int..

[24] Melendez G.C., Li J., Law B.A., Janicki J.S., Supowit S.C., Levick S.P. (2011). Substance P induces adverse myocardial remodelling via a mechanism involving cardiac mast cells. Cardiovasc. Res..

[25] Weglicki W.B., Mak I.T., Phillips T.M. (1994). Blockade of cardiac inflammation in Mg2+ deficiency by substance P receptor inhibition. Circ. Res..

[26] Mak I.T., Chmielinska J.J., Kramer J.H., Spurney C.F., Weglicki W.B. (2011). Loss of neutral endopeptidase activity contributes to neutrophil activation and cardiac dysfunction during chronic hypomagnesemia: Protection by substance P receptor blockade. Exp. Clin. Cardiol..

[27] Munoz M., Covenas R. (2020). The Neurokinin-1 Receptor Antagonist Aprepitant: An intelligent bullet against cancer?. Cancers.

[28] Munoz M., Covenas R. (2013). Involvement of substance P and the NK-1 receptor in cancer progression. Peptides.

[29] Munoz M., Covenas R., Esteban F., Redondo M. (2015). The substance P/NK-1 receptor system: NK-1 receptor antagonists as anti-cancer drugs. J. Biosci..

[30] Munoz M., Covenas R. (2010). Neurokinin-1 receptor: A new promising target in the treatment of cancer. Discov. Med..

[31] Munoz M., Gonzalez-Ortega A., Covenas R. (2012). The NK-1 receptor is expressed in human leukemia and is involved in the antitumor action of aprepitant and other NK-1 receptor antagonists on acute lymphoblastic leukemia cell lines. Invest. N. Drugs.

[32] Munoz M., Berger M., Rosso M., Gonzalez-Ortega A., Carranza A., Covenas R. (2014). Antitumor activity of neurokinin-1 receptor antagonists in MG-63 human osteosarcoma xenografts. Int. J. Oncol..

[33] Munoz M., Gonzalez-Ortega A., Salinas-Martin M.V., Carranza A., Garcia-Recio S., Almendro V., Coveñas R. (2014). The neurokinin-1 receptor antagonist aprepitant is a promising candidate for the treatment of breast cancer. Int. J. Oncol..

[34] Munoz M., Covenas R. (2015). Targeting NK-1 receptors to prevent and treat pancreatic cancer: A new therapeutic approach. Cancers.

[35] Cardinale D., Colombo A., Bacchiani G., Tedeschi I., Meroni C.A., Veglia F., Civelli M., Lamantia G., Colombo N., Curigliano G. (2015). Early detection of anthracycline cardiotoxicity and improvement with heart failure therapy. Circulation.

[36] Zamorano J.L., Lancellotti P., Rodriguez Munoz D., Aboyans V., Asteggiano R., Galderisi M., Habib G., Lenihan D.J., Lip G.Y.H., Lyon A.R. (2017). 2016 ESC Position Paper on cancer treatments and cardiovascular toxicity developed under the auspices of the ESC Committee for Practice Guidelines: The Task Force for cancer treatments and cardiovascular toxicity of the European Society of Cardiology (ESC). Eur. J. Heart Fail..

